# Rapid Presentation of Emotional Expressions Reveals New Emotional Impairments in Tourette’s Syndrome

**DOI:** 10.3389/fnhum.2013.00149

**Published:** 2013-04-24

**Authors:** Martial Mermillod, Damien Devaux, Philippe Derost, Isabelle Rieu, Patrick Chambres, Catherine Auxiette, Guillaume Legrand, Fabienne Galland, Hélène Dalens, Louise Marie Coulangeon, Emmanuel Broussolle, Franck Durif, Isabelle Jalenques

**Affiliations:** ^1^Université Grenoble Alpes, LPNC, Grenoble and CNRS, LPNC UMR 5105Grenoble, France; ^2^Institut Universitaire de FranceParis, France; ^3^Laboratoire de Psychologie Sociale et Cognitive, Clermont Université, Université Blaise Pascal and UMR 6024 CNRSClermont-Ferrand, France; ^4^Service de Neurologie A, CHU Gabriel MontpiedClermont-Ferrand, France; ^5^Pôle de Psychiatrie, Service de Psychiatrie de l’Adulte A et Psychologie Médicale, CHU Clermont-FerrandClermont-Ferrand, France; ^6^UFR Médecine, Clermont Université, Université d’Auvergne Clermont 1Clermont-Ferrand, France; ^7^Service de d’Ophtalmologie, CHU Gabriel MontpiedClermont-Ferrand, France; ^8^Service de Neurologie C, Hôpital Neurologique de LyonLyon, France

**Keywords:** Tourette’s syndrome, emotional facial expressions, embodiment theory, spatial frequency analysis

## Abstract

**Objective:** Based on a variety of empirical evidence obtained within the theoretical framework of embodiment theory, we considered it likely that motor disorders in Tourette’s syndrome (TS) would have emotional consequences for TS patients. However, previous research using emotional facial categorization tasks suggests that these consequences are limited to TS patients with obsessive-compulsive behaviors (OCB).

**Method:** These studies used long stimulus presentations which allowed the participants to categorize the different emotional facial expressions (EFEs) on the basis of a perceptual analysis that might potentially hide a lack of emotional feeling for certain emotions. In order to reduce this perceptual bias, we used a rapid visual presentation procedure.

**Results:** Using this new experimental method, we revealed different and surprising impairments on several EFEs in TS patients compared to matched healthy control participants. Moreover, a spatial frequency analysis of the visual signal processed by the patients suggests that these impairments may be located at a cortical level.

**Conclusion:** The current study indicates that the rapid visual presentation paradigm makes it possible to identify various potential emotional disorders that were not revealed by the standard visual presentation procedures previously reported in the literature. Moreover, the spatial frequency analysis performed in our study suggests that emotional deficit in TS might lie at the level of temporal cortical areas dedicated to the processing of HSF visual information.

## Introduction

### Tourette’s syndrome: Physiopathology and emotional disorders

Tourette’s syndrome (TS) is a neuropsychiatric syndrome defined by multiple chronic motor and vocal tics. Moreover, TS is a pathology associated with a high occurrence of comorbid conditions (Robertson, 2000). The most common are attention-deficit/hyperactivity disorder (ADHD) and obsessive-compulsive behaviors (OCB) (Cavanna et al., 2009; Worbe et al., 2010). Concerning the physiopathology of TS, Mink (2001) suggested that TS probably involves complex basal ganglia and frontocortical circuits (but, see also Osmon and Smerz, 2005, for a review). Albin and Mink (2006) proposed the hypothesis of a subcortical hyperdopaminergy in TS, based on biochemical analyses of postmortem striatum from TS patients which revealed a significant increase in the number of dopamine uptake carrier sites (Singer and Walkup, 1991; Singer et al., 1991) at the level of both striatal (Wong et al., 1997) and cortical structures (Yoon et al., 2007). This hypothesis has found support from experiments involving animals which have shown that the injection of a dopaminergic agonist increases the production of motor stereotypy in a way very similar to that observed in TS (Delfs and Kelley, 1990; Palminteri et al., 2011). Moreover, medication and imaging studies, combined with analyses of human materials involving blood, urine, cerebrospinal fluid, and postmortem brain tissue, suggest the existence of other neurochemical deviances in TS which relate to the dopaminergic system, as well as to the serotoninergic, noradrenergic, glutamatergic, Gamma-aminobutyric acid (GABA)-ergic, cholinergic, and opioid metabolism (Harris and Singer, 2006). It has long been suspected that such imbalances would be observed due to the interaction between the serotonergic and dopaminergic systems (Steeves et al., 2010). It has been assumed not only that these hyperdopaminergic disorders induce significant motor dysfunctions (involving both verbal and motor tics) but also that they may have important consequences at the emotional level.

In addition to the possibility of a potential emotional deficit in TS due to dopaminergic modulation at the level of the central nervous system, recent studies falling within the general framework of embodiment theory suggest that motor disorders at a peripheral level could have major consequences for both cognitive (Boulenger et al., 2008) and emotional processes (Dimberg, 1990; Niedenthal, 2007; Niedenthal et al., 2010; Beffara et al., 2012). Based on the experimental evidence resulting from embodiment theory, we assume that motor disorders (motor or verbal tics) might produce different impairments on emotional facial expressions (EFEs). Recent articles have shown that this sensorimotor integration of emotional stimuli could occur in a very fast and automatic manner (Vermeulen et al., 2009a,b). Emotional impairments on EFE can therefore occur in response to the fast presentation of emotional stimuli.

A number of studies have been undertaken in order to investigate the possibility of emotional disorders in TS. Among these different studies, those performed by Sprengelmeyer et al. (1996) and Sprengelmeyer et al. (1997) have led to the proposal that the neurophysiological and neuropsychological data obtained from subjects with obsessive-compulsive disorder (OCD) point to abnormalities in fronto-striatal regions (Abbruzzese et al., 1997) that may potentially mediate correct categorization of EFEs of disgust. Thus, Sprengelmeyer et al. (1997) found that TS patients with OCB have a specific impairment on disgusted faces. The same study did not reveal any impairment in TS patients without OCB. Similar results were obtained in fMRI experiments conducted by Shapira et al. (2003). These results indicated that the patterns of brain activation in OCD patients and control participants differed after disgust-inducing visual stimulation, whereas activation after a threat-inducing stimulation was similar in the two groups. However, in an attempt to replicate Sprengelmeyer et al. (1997), Parker et al. (2004) failed to reproduce this finding with OCD patients and observed an impairment of disgust in one specific OCD patient only. However, we consider that the task sensitivity in these different studies may not have been sufficient to reveal clear evidence of *emotional* impairments. In both Sprengelmeyer et al. (1997) (TS patients with or without OCB and OCD patients) and Parker et al. (2004) (OCD patients), participants were exposed to morphed stimulation an emotion hexagon taken from Ekman and Friesen’s (1976) database. The advantage of this approach lies in the fact that participants are repeatedly exposed to a continuous variation of the same EFE. However, a potential disadvantage is that this type of repeated exposure to the stimuli across the hexagon significantly improves the participant’s ability to categorize the EFEs and this might be sufficient to induce ceiling effects based on a purely perceptual analysis of the stimuli (Niedenthal et al., 2010). In fact, when long and repeated exposure durations (1000–5000 ms stimulus onset) are used, participants may employ a perceptual analysis based on specific features to perform the task (Kaminski et al., 2010, 2011). For example, Adolphs et al. (2005) showed that a patient with a lesion of the amygdala who was no longer sensitive to fearful expressions was nonetheless able to categorize fearful expressions by looking at specific perceptual details such as the opening of the eyes. This experiment illustrates the fact that participants may use perceptual details to perform the classification task, *even if they have no clear emotional feeling for the different EFEs*. We therefore decided to use a very fast presentation of the stimuli (200 ms onset) in order to prevent the explicit use of perceptual analysis when performing the task. We consider that this type of rapid presentation might reduce the use of perceptual strategies of the sort that might have been employed in other studies where they could have compensated for specific emotional deficits which went undetected due to the longer presentation durations.

### Preferential link between LSF information and emotional processes

In addition to these global emotional deficits that are possibly induced by motor disorders, potential subcortical dysfunctions in TS may have other consequences for specific emotional processes that occur at the level of the basal ganglia. The subcortical hyperdopaminergy assumed to occur at the level of the basal ganglia in TS might induce a disturbance of subcortical limbic integration. This type of subcortical disturbance may have functional consequences for the integration of the low spatial frequency information associated with emotional stimuli. Various neuroimaging (Vuilleumier et al., 2003; Pourtois et al., 2005), behavioral (Bocanegra and Zeelenberg, 2009; Mermillod et al., 2010b), and neurocomputational results (Mermillod et al., 2009b, 2010a) point to a preferential link between low spatial frequency information and subcortical structures dedicated to emotional processing. In the lateral geniculate nucleus, the magnocellular layers act as a high-pass temporal frequency filter and low-pass spatial frequency (LSF) filter, whereas the parvocellular layers correspond to a low-pass temporal filter which processes high spatial frequency (HSF) information. Given our knowledge of retinal projections, this preferential link could take the form of a “quick and dirty” perceptual pathway to subcortical areas (Ledoux, 1996; de Gelder et al., 1999; Pegna et al., 2004). However, even if this type of direct pathway has been found to exist in archaic mammals such as rats (Ledoux, 1996), the question of whether it also exists in humans is as yet unresolved (Mermillod et al., 2011). An alternative hypothesis holds that these subcortical structures are rapidly activated through fast track projections of magnocellular layers to frontal areas dedicated to the top-down guidance of visual perception (Bullier, 2001; Bar, 2004; Peyrin et al., 2006, 2010). It has also recently been suggested that a similar preferential link exists between LSF information and emotional processes in the form of the top-down regulation of subcortical structures (Kveraga et al., 2007). We therefore assume here that there is a preferential link between the LSF information and the emotional processes that take place in the various subcortical structures that are potentially involved in TS. More precisely, we assume that LSF emotional information might be particularly impaired by fronto-striatal abnormalities (Abbruzzese et al., 1997) or by the hyperdopaminergy of subcortical structures (Singer and Walkup, 1991; Singer et al., 1991; Albin and Mink, 2006).

### Hypotheses

We assumed that it might be possible to reveal widespread emotional deficits in TS by presenting the stimuli rapidly in order to reduce the use of perceptual strategies that may potentially compensate for emotional disorders. Moreover, based on the above-mentioned literature reporting a preferential link between subcortical emotional processes and LSF visual information, we assume that these emotional impairments might be observed more specifically on the LSF than on the HSF components of EFE. The average categorization rate of TS patients was compared with that of matched healthy controls (MHC).

## Materials and Methods

### Participants

The study protocol was approved by the regional Medical School Ethics Committee (AU701) and the study was conducted in accordance with the principles set out in the Declaration of Helsinki and with French legislation (the Huriet law). The nature and potential risks of the study were fully explained and written informed consent was obtained from each patient and MHC participant. The study was also registered with the clinical trial-specific website (NCT: 00664300).

#### Tourette’s syndrome patients

Patients were included if they corresponded to a DSM-IV-R diagnosis of TS, stabilized for at least 1 month, and were receiving medical treatment. They were recruited during a multidisciplinary consultation at the Clermont-Ferrand and Lyon university hospitals. The diagnoses of TS and comorbidities were established by two experienced neurologists and psychiatrists. Patients with other comorbidities described below were excluded from the experiment.

#### Matched healthy controls

In order to compare the performance of TS patients in the categorization of EFEs, these patients were paired with 18 healthy controls matched with them for age, gender, and educational level (Table 1). MHC aged 18–70 years (*M* = 33.9, SD = 16.1), were recruited. The results of neuropsychological, psychiatric, visual perception, and psychological tests revealed normal scores in all MHC.

**Table 1:** **Characteristics of Tourette’s syndrome (TS) and matched healthy control (MHC) participants**.

Sex	TS with OCD (9)		Matched healthy controls (9)		TS without OCD (9)		Matched healthy controls (9)	
	Female (3)	Male (6)	Female (3)	Male (6)	Female (3)	Male (6)	Female (3)	Male (6)
	Mean	SD	Mean	SD	Mean	SD	Mean	SD
Age	28.67	12.85	28.78	13.44	37.33	17.71	39.00	17.61
EL	2.33	1.41	2.89	1.62	2.56	1.24	2.22	1.20
MMS	29.00	1.00	29.89	0.33	29.78	0.44	30.00	0.00

*EL, educational level; MMS, mini mental state*.

### Neurological and psychiatric assessments

The severity of the tics was evaluated using the Yale Global Tic Severity Scale (YGTSS) (Leckman et al., 1989) and the TS patients completed a number of psychological, psychiatric, visual perception, and neuropsychological tests as described below. The aim was to ensure that potential deficits in the emotional categorization tasks were not due to perceptual or mood disorders, IQ, prosopagnosia, or ADHD.

#### Psychiatric comorbidities

In order to characterize the comorbid psychiatric conditions that are most commonly encountered in TS (Cavanna et al., 2009), all the patients were evaluated using psychiatric self-rating scales, a semi-structured interview administered by trained psychiatrists, the French version of the Mini International Neuropsychiatric Interview (M.I.N.I.; version 5.0.0) (Sheehan et al., 1998) and a clinical diagnosis based on DSM-IV-R criteria.

#### Psychiatric self-rating scales

With regard to the potential ADHD, we first evaluated the patients using the Copeland Symptom Checklist for Adult Attention-Deficit Disorders (Copeland, 1989) modified for young adults and adults. To examine potential bipolar comorbidity, we first evaluated each patient on the basis of the Mood Disorder Questionnaire (MDQ) (Hirschfeld et al., 2003; Weber Rouget et al., 2005). The other self-rating scales were the Hospital Anxiety and Depression Scale (HAD) for anxiety and depressive disorders (Zigmond and Snaith, 1983; Lepine et al., 1986) and Pauls’ questionnaire (derived from the Yale–Brown obsessive-compulsive scale, Y-BOCS, Goodman et al., 1989) for repetitive behaviors and thoughts.

#### Psychiatric interview

The final assessment of potential comorbidities was performed for each patient on the basis of the M.I.N.I. version 5.0.0 and an individual diagnosis established by a psychiatrist according to DSM-IV-R criteria (American Psychiatric Association, 2003). The impact of the symptoms on social and occupational functioning was evaluated using the Global Assessment of Functioning scale (GAF) which is the fifth axis of the DSM-IV-R.

We also assessed the patients for ADHD, OCD (based on Pauls’ questionnaire), anxiety and mood disorders based on the HAD and MDQ questionnaire, and tic severity (YGTSS). Both self-rating scales and an interview with a psychiatrist were necessary in order to classify the different types and lifetime appearances of obsessions or compulsions from which TS patients suffer (Worbe et al., 2010).

#### Obsessive-compulsive disorder/obsessive-compulsive behaviors

Repetitive behaviors and thoughts were identified using the M.I.N.I. version 5.0.0, a semi-directed psychiatric interview (Worbe et al., 2010) and a self-rated questionnaire derived from the Yale–Brown obsessive-compulsive scale (Y-BOCS, Goodman et al., 1989).

### Neuropsychological assessments

#### Wechsler adult intelligence scale

First of all, IQ was assessed using the Wechsler Adult Intelligence Scale (WAIS-III, Wechsler, 1989) in order to evaluate global and aggregate cognitive functioning via verbal IQ, performance IQ, and full scale IQ (verbal comprehension, working memory, perceptual organization, and processing speed).

#### Mini mental state

A Mini Mental State (MMS, Folstein et al., 1975) was administered to assess mental and perceptive functions (e.g., memory, spatial and time orientations, attention), principally to exclude dementia.

#### Attentional disorders

Brickenkamp and Zillmer (1998) D2 and classic Stroop task (Stroop, 1935) were administered in order to assess for potential attentional disorders in the TS patients. The Stroop task provided an indicator of attention and learning disorders whereas the D2 test informed us about the fatigability, speed, and efficiency of information processing.

#### Benton’s facial recognition test

In order to assess for potential problems relating to prosopagnosia in the TS patients or MHC, we administered a Benton Facial Recognition Test. In the Benton Facial Recognition Test (Levin et al., 1975), participants are asked to recognize non-familiar faces in different lighting conditions and viewed from different angles. This task requires the integration of visuospatial abilities.

#### Visual perception test

Because we were investigating the ability to perform emotional categorization in different SF channels, we administered a VISTECH visual perception test to ensure that neither TS patients nor MHC were suffering from any visual deficit in specific SF channels. VISTECH is a standard test of sensitivity to contrast and spatial frequency in which participants are asked to determine the orientation of lines filtered at different spatial frequencies.

#### Understanding of emotion

Finally, to assess whether the participants correctly understood emotional words, we administered a task requiring the semantic recognition of such words (Sprengelmeyer et al., 1997). This test was presented in order to determine whether the patients were able to define the meaning of the six basic emotions correctly by specifying a situation in which the corresponding emotion would be felt.

### Stimuli and material

The participants were tested individually in a shielded room where they sat at a viewing distance of 90 cm in front of a 19″ CRT ViewSonic^®^ monitor connected to a PC. The experimental events were controlled by E-Prime^®^ 1.2 (Psychology Software Tools, Pittsburg, PA, USA).

The images were taken from Ekman and Friesen’s (1976) database. Each face of a different individual (five male and five female) corresponded to one of six basic emotional expressions (anger, disgust, fear, happiness, sadness, surprise) or a neutral expression. These 70 black-and-white photographs were either unfiltered (BSF, broad spatial frequencies) or modified by means of two filters applied using Matlab software: a low-pass filter for low spatial frequencies (LSF, <6 cycles/image), and a high-pass filter for high spatial frequencies (HSF, >24 cycles/image). These thresholds were chosen in the light of previous articles showing a clear differentiation of emotional processes for these values (Schyns and Oliva, 1999; Vuilleumier et al., 2003; Mermillod et al., 2009a, 2010a,b). All stimuli were normalized for luminance and contrasts did not differ statistically between experimental conditions. The 210 stimuli (7 EFEs × 10 individuals × 3 SF levels) were presented in the center of the computer screen and randomized for the experimental session.

### Procedure

In order to make it less likely that the participants would be able to use perceptual strategies to categorize the EFE, we employed a task in which they had to categorize EFEs that were presented only briefly. After a fixation cross had been presented for 5 s, an EFE was immediately displayed in the center of the computer screen for 200 ms. Each face was followed by a 30 ms mask to avoid retinal persistence. The participants performed a 12-trial training session in order to ensure that they correctly understood the aim of the task. This training session was followed by the experimental session which consisted of 210 trials. In response to each picture, and subject to no time constraints, the participants had to state the name of the EFE, which they categorized as one of the six basic emotions or as a neutral expression. To help them, they could see a card on which the names of the seven possible responses were written. Because of the motor disorders present in TS, the experimenter either pressed one of the seven keys corresponding to their responses or recorded the fact that the participant had not perceived the face at all. Moreover, because of potential attention disorders in TS, a screen was displayed before each trial and the participant pressed the space bar to move on to the next trial only when he or she was ready to do so.

### Statistical analysis

We conducted an analysis of variance (ANOVA) on the ratio of correct responses for both the TS and MHC groups. We used a between-group repeated-measures ANOVA with the factors “EFEs” (Anger vs. Disgust vs. Fear vs. Happiness vs. Neutrality vs. Sadness vs. Surprise) and “Spatial Frequencies” (BSF; HSF; LSF) as within-subjects variables and Experimental Group (TS patients vs. MHC participants) as between-subject variable. In order to correct a violation of the sphericity assumption (inhomogeneity of covariance) for within-subject variables such as Emotion and Emotion × Group, we applied a Huynh–Feldt adjustment. Next, again due to the violation of sphericity, we conducted non-parametric analyses for local comparisons. Consequently, the categorization performance of the TS patients was compared with that of MHC using a two-tailed Mann–Whitney *U* test. We used Statistica^®^ 7.0 and IBM SPSS Statistics^®^ 20 software for the statistical analysis.

## Results

First, we verified that age, sex, and level of education did not statistically differ between MHC and TS patients.

### Neurological and psychiatric assessments

Eighteen TS patients (12 men, 6 women) aged 16–68 years (*M* = 33.0, SD = 15.7) were included. The results of the MMS revealed normal scores for all TS patients (Table 1). The Copeland Symptom Checklist revealed that all the items were normal except for Inattention/Distractibility (*M* = 42.1, SD = 24.7) for which minor to moderate difficulties were found. Psychiatric comorbidities are presented in Table 2. Nine patients exhibited both TS and OCD and the different types and lifetime appearance of obsessions or compulsions from which TS patients suffer are presented in the result section. With regard to the impact of the symptoms on social and occupational functioning evaluated using the GAF, the TS patients’ scores (*M* = 52.8, SD = 8.54) revealed an average level of difficulty (Table 3).

**Table 2:** **Details of Tourette’s syndrome (TS) comorbidities**.

TS N°	GAF	Disorders						
		Anxiety		OCD	Mood		Addictions	
1	61	–	–	No	–	–	–	–
2	70	–	–	No	MDD	Past	Alcohol	Past
3	61	–	–	No	–	–	–	–
		Agoraphobia	Past and present
4	61	Social phobia	Past and present	No	MDD	Past	–	–
		GAD	Past and present	
5	48	Agoraphobia	Past and present	No	Suicide risk (light)	Present	Alcohol	Present
6	50	–	–	No	–	–	–	–
7	50	–	–	No	–	–	–	–
8	41	Social phobia	Past	No	–	–	–	–
9	–			No	Suicide risk (high)	Present	Cannabis	Past
10	51	Personality	Past and present	Yes	Suicide risk (light)	Present
					Hypomanic	Past	–	–
11	49	GAD	Past	Yes	MDD	Past	–	–
					MDD	Present
12	50	–	–	Yes	Dysthymia	Present	–	–
					Manic episode	Past	
13	51	Agoraphobia	Past and present	Yes	–	–	–	–
14	41	GAD	Past	Yes	MDD	Past	Alcohol	Past
15	45	GAD	Present	Yes	Suicide risk (light)	Present	–	–
16	–	–	–	Yes	–	–	–	–
17	–	GAD	Present	Yes	MDD	Past	–	–
18	60	Agoraphobia	Present	Yes	–	–	–	–
		GAD	Present	

*GAD, generalized anxiety disorder; OCD, obsessive-compulsive disorders; MDD, major depressive disorder*.

**Table 3:** **Summary of scores on psychiatric and neuropsychological assessments**.

			TS with OCD (9)		TS without OCD (9)		TS (18)	
			Female (3)	Male (6)	Female (3)	Male (6)	Female (6)	Male (12)
			Mean	SD	Mean	SD	Mean	SD
		Age	28.7	12.8	37.3	17.7	33.0	15.7
		Wais-III	98.4	14.1	102	15.3	100	14.4
		MMS	29.8	0.4	29.0	1.0	29.4	0.8
		GAF	49.6	5.88	55.3	9.47	52.8	8.26
**PSYCHIATRIC ASSESSMENTS**								
	**Copeland**							
		Inattention/Distractibility	52.4	22.2	31.7	23.7	42.1	24.7
		Impulsiveness	44.8	26.5	32.6	16.0	38.7	22.1
		Overactivity/hyperactivity	45.7	24.8	26.5	15.2	36.1	22.2
		Underactivity	37.3	19.8	26.5	19.6	31.9	19.9
		Under-achievement/disorganization/learning disorder	36.7	23.5	22.9	9.9	29.8	18.9
**NEUROPSYCHOLOGICAL ASSESSMENTS**								
	**D2**							
		GZ	419	172	426	61.0	448	70.2
		*F*%	14.3	19.9	20.6	14.7	18.5	17.2
		GZ – F	468	66.6	406	67.4	435	72.4
		KL	165	74.9	156	31.3	170	36.7
		SB	12.2	6.0	12.2	4.3	12.9	4.2
	**Stroop**
		Reading 1	95.3	13.8	98.4	4.7	96.9	10.1
		Reading 2	90.6	14.6	87.7	13.5	89.1	13.7
		Spelling	70.8	14.5	75.7	10.2	73.2	12.4
		Interference	42.4	10.2	39.2	11.0	40.8	10.4
		Overall score	28.3	11.6	36.4	14.0	32.4	13.1
		Benton	46.4	4.4	42.4	7.3	44.4	6.2

*TS, Tourette’s Syndrome; MHC, matched healthy controls; OCD, obsessive-compulsive disorders; MMS, mini mental state; GAF, global assessment of functioning; WAIS, Wechsler adult intelligence scale*.

### Neuropsychological assessments

The patients obtained normal IQ scores of between 90 and 109 (*M* = 100.2, SD = 14.4). Consequently, we did not exclude any patient on the basis of IQ deficiency. As far as MMS is concerned, a score lower than 26 (/30) was an exclusion criterion (*M* = 29.4, SD = 0.850) (see Table 3). However, as for IQ, no patients were excluded due to low MMS scores. With regard to the D2 task, the results revealed impaired attention characterized by uncontrolled errors (*F*% = 18.5). Nevertheless, this was not a function of either stimulation (GZ = 447) or concentration (KL = 170). We did not exclude any patients due to abnormal attention disorders and the Stroop task confirmed that no such exclusions were required (Table 3). Moreover, TS patients with OCD did not statistically differ from patients without OCD.

The scores obtained by the TS patients on Benton’s Facial Recognition Test did not differ statistically from those of the MHC (*M* = 44.4, SD = 6.20) (see Table 3). However, we excluded two TS patients (over 20 patients) due to visual deficiencies revealed by the VISTECH, whereas all MHC participants had normal or corrected-to-normal vision. Finally, we did not exclude any TS patients or MHC due to an incorrect understanding of emotional words.

### Correct categorization rate

#### Analysis of variance

**Figure 1:**
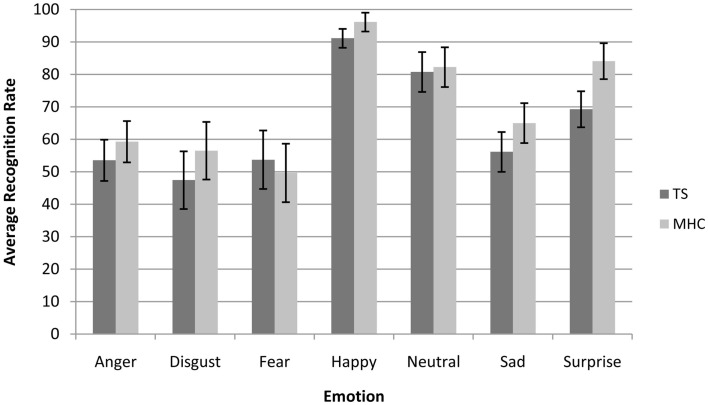
**Average recognition rate (collapsed across all spatial frequencies) of Tourette’s patients compared to matched healthy control participants for each emotional expression**. Bars represent 95% of confidence interval.

A canonical analysis revealed a significant main effect of Emotion, *F*(6, 204) = 54.4, *p* < 0.001, ${\text{η}}_{\text{p}}^{\text{2}}$ = 0.615, which resulted in a better categorization of specific emotions, for instance happiness, compared to other emotions such as those conveyed by the expressions of fear or disgust (Figure 1).

Moreover, and in accordance with the literature on presentation times above 50 ms (Schyns and Oliva, 1994), we also observed a significant main effect of spatial frequency channels, *F*(2, 68) = 89.5, *p* < 0.001, ${\text{η}}_{\text{p}}^{\text{2}}$ = 0.725, with the result that the participants were more accurate on the BSF faces than the HSF faces and more accurate on the HSF than on the LSF faces. We did not observe a main effect of experimental group *F*(1, 34) = 2.65, *p* = 0.110, ns. However, as was expected on the basis of our hypotheses, we observed a marginally significant three-way Emotion × Spatial Frequencies × Experimental Group interaction, *F*(12, 408) = 1.83, *p* = 0.05, ${\text{η}}_{\text{p}}^{\text{2}}$ = 0.051. In line with the hypotheses stated above, we investigated the possibility that differences between TS and MHC are probably restricted to specific emotions and spatial frequency channels (Table 4).

**Table 4:** **Average correct recognition rate for TS patients and MHC participants for each emotion and spatial frequency channel**.

	BSF faces		HSF faces		LSF faces	
	TS	MHC	TS	MHC	TS	MHC
Anger	61.7*	75.0	58.9	64.4	40.0	38.3
Disgust	58.9	66.1	52.2	60.6	31.1	42.8
Fear	67.2	54.4	53.9	47.8	40.0	46.7
Happiness	93.3	96.7	92.2	95.6	87.8	96.1
Neutrality	81.1	83.9	86.7	86.7	74.4	76.1
Sadness	66.1	71.1	60*	74.4	42.2	49.4
Surprise	72.2*	88.3	66**	84.4	70.0	79.4

*p < 0.05, **p < 0.01.

*BSF, broad spatial frequencies; HSF, high spatial frequencies; LSF, low spatial frequencies*.

#### Non-parametric tests

Local comparisons of TS and MHC were performed using a two-tailed Mann–Whitney *U* test because the assumption of homogeneity of covariance necessary to perform an ANOVA was violated for several comparisons. These comparisons revealed that, in the case of the BSF faces, TS patients exhibited significantly more impairments than MHC in response to the expressions of anger, *U* = 235, *p* = 0.02, *r* = 0.393 and surprise, *U* = 257, *p* = 0.002, *r* = 0.513. The differences were not significant for any of the other BSF EFEs. As far as the HSF faces are concerned, the statistical analyses showed that TS patients were significantly more impaired than MHC on the expressions of sadness, *U* = 227, *p* = 0.04, *r* = 0.348 and surprise, *U* = 255, *p* = 0.003, *r* = 0.498. The differences were not significant for any of the other HSF EFEs or any of the LSF faces (Table 4).

### Confusion matrices

**Figure 2:**
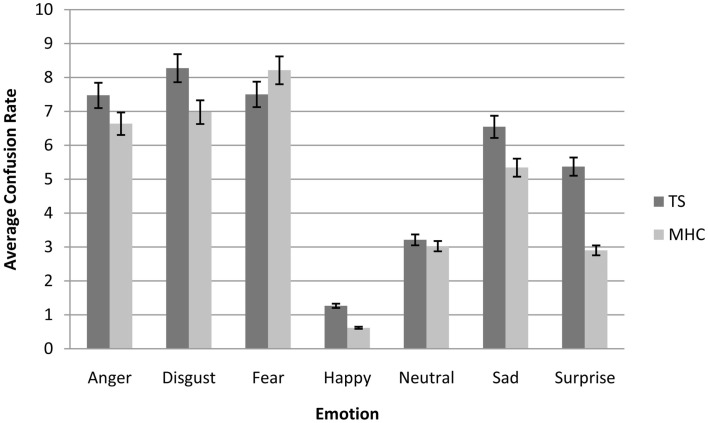
**Average confusion matrices (collapsed across all spatial frequencies) of Tourette’s patients compared to matched healthy control participants for each emotional expression**. Bars represent 95% of confidence interval.

In order to understand how these misclassifications occurred in the TS patients, we computed an average confusion rate for TS and MHC participants. As shown in Figure 2, the confusion matrices suggest that the lower categorization rate of TS patients was accompanied by a misclassification of other emotions, but not with an overestimation in the recognition of neutral expressions. In order to perform a simple statistical analysis of the results of the confusion matrices, we computed an average level of misclassification (i.e., the average number of responses citing an EFE other than the actually displayed emotion) for each EFE and each spatial frequency (Table 5).

**Table 5:** **Average confusion rate for TS patients and MHC participants for each emotion and spatial frequency channel**.

	BSF faces		HSF faces		LSF faces	
	TS	MHC	TS	MHC	TS	MHC
Anger	6.3*	4.17	6.57	5.83	9.54	9.91
Disgust	6.39	5.56	7.50	6.11	10.93	9.26
Fear	5.37	7.41	7.69	8.43	9.44	8.80
Happiness	0.93	0.56	1.20	0.74	1.67	0.56
Neutrality	3.06	2.50	2.04	2.13	4.54	4.44
Sadness	5.00	4.35	5.93	3.80	8.70	7.87
Surprise	5.28**	2.13	5.93*	3.06	4.91	3.52

*p < 0.05, **p < 0.01.

*BSF, broad spatial frequencies; HSF, high spatial frequencies; LSF, low spatial frequencies*.

#### Analysis of variance

In the same way as for the correct categorization rate, the canonical analysis revealed a significant main effect of Emotion, *F*(6, 204) = 47.6, *p* < 0.001, ${\text{η}}_{\text{p}}^{\text{2}}$ = 0.72, resulting in more confusions for specific emotions (Figure 2). We also observed a significant main effect of spatial frequency channels, *F*(2, 68) = 82.3, *p* < 0.001, ${\text{η}}_{\text{p}}^{\text{2}}$ = 0.41, which resulted in less confusion for BSF faces than for HSF faces and less confusion for HSF than for LSF faces. As was the case for accuracy, we did not observe a main effect of experimental group, *F*(1, 34) = 2.14, *p* = 0.15, *ns* nor any significant interactions between experimental group and EFE or spatial frequencies. This was possibly due to specific, non-generalized deficits in TS on particular emotions and spatial frequency channels (Table 5).

#### Non-parametric tests

Because there was no systematic homogeneity of covariance, we used a two-tailed Mann–Whitney *U* test to conduct local comparisons between TS and MHC. These comparisons revealed that in the case of BSF filtering, TS patients produced significantly more confusions with other EFEs than MHC following the presentation of angry, *U* = 94, *p* = 0.031, *r* = 0.366 and surprise expressions, *U* = 64, *p* = 0.0014, *r* = 0.530. The differences were not significant for any of the other BSF EFEs. As far as the HSF faces are concerned, the statistical analyses showed that TS patients exhibited a significantly higher level of confusion than MHC when required to categorize EFEs of surprise, *U* = 70, *p* = 0.003, *r* = 0.493. The differences were not significant for any of the other HSF EFEs or any of the LSF faces.

### Tic severity and emotional impairments

In order to test the possibility of a relationship between the severity of the tics and emotional impairment in the recognition of facial expressions, we applied a median split (median value = 37) to the YGTSS results. The ANOVA on the factors “EFEs” and “Spatial Frequencies” as within-subjects variables and Experimental Group (low YGTSS patients vs. high YGTSS patients) as between-subject variable revealed no significant differences for EFE, SF, Experimental Group, or any significant interactions among these factors.

### Obsessive-compulsive disorders and emotional impairments

In the light of Sprengelmeyer et al.’s (1997) results, we introduced OCD as a factor in the statistical analysis on the basis of the participants’ self-rated obsession and compulsion questionnaires. For the same reasons as mentioned above, we first conducted an ANOVA with a Huynh–Feldt adjustment for within-subject variables (Emotion and SF) and a non-parametric analysis for local comparisons.

Nine TS patients with OCD were compared with nine TS patients without OCD in terms of their ability to correctly categorize EFE. An ANOVA corrected for non-sphericity revealed that the OCD factor (TS with OCD vs. TS without OCD) was significant, *F*(1, 16) = 5.08, *p* < 0.05, ${\text{η}}_{\text{p}}^{\text{2}}$ = 0.241. Surprisingly, however, TS without OCD were less accurate (*M* = 0.588, SE = 0.360) than TS with OCD (*M* = 0.703, SE = 0.360). This effect was confirmed by a non-parametric analysis revealing a significant main effect of the OCD factor, *U* = 64.0, *p* < 0.05, *r* = 0.490. Compared to TS with OCD, TS without OCD exhibited a lower EFE categorization rate. In order to determine which emotional expression was impaired, a two-tailed Mann–Whitney *U* test was run and revealed that this effect was due only to a significant difference on disgusted faces. Indeed, TS patients without OCD exhibited significant impairments compared to TS patients with OCD when required to recognize disgust in HSF faces, *U* = 75.0, *p* = 0.001, *r* = 0.728 and LSF faces, *U* = 63.5, *p* = 0.04, *r* = 0.486. The differences were not significant for any other BSF, HSF, or LSF EFEs.

## Discussion

Our current results indicate that, once the potential use of cognitive strategies based on a perceptual analysis of the images had been restricted, TS patients produced significant impairments in response to the EFEs of anger and surprise in unfiltered BSF faces. Moreover, the results revealed a significantly lower level of recognition of sadness and surprise in the HSF channels but no impairments on LSF faces. In addition, it is possible that the same trend observed for other emotional expressions (the happy and disgusted EFEs, in particular) might not be significant due to a lack of power or because our task, even though it reduced the possible use of *perceptual* strategies, was not sufficiently sensitive to *emotional* deficits. These results are consistent with recent papers revealing specific disorders in theory of mind in TS patients (Eddy et al., 2010).

Since HSF are processed by temporal cortical areas, these results suggest that the locus of the potential dysfunctioning of emotional processes in TS may be found at the level of temporal cortical areas rather than subcortical structures. This possibility is consistent with the neural basis of embodiment theory (Niedenthal et al., 2010) which emphasizes the importance of the cortical integration of motor behavior in achieving a correct understanding of emotional stimuli. The idea that embodiment processes might contribute to these lower recognition rates is further supported by the analysis of the confusion matrices which shows that the decrease in categorization accuracy is not due to an increase in the recognition of neutral expressions, as might be the case with facial amimia in parkinsonian patients (Mondillon et al., 2012) but rather to a mismatch with other emotions, as the presence of tics in TS suggests. Unfortunately, the small sample of patients compared on the basis of the YGTSS did not allow us to confirm this relationship between the severity of tics and the severity of impairment of EFE recognition. This hypothesis has to be carefully addressed in future studies based on the electromyographic recording of facial muscles during the evaluation of emotional capacities.

We also observed a specific deficit on disgusted faces which was limited to TS without OCD only. These results reveal striking differences with previous papers on this topic involving TS patients (Sprengelmeyer et al., 1997). It seems probable that this discrepancy is due to important methodological differences between previous experiments and our current study. The results reported by Sprengelmeyer et al. (1997) used a longer presentation time (5000 ms onset) while we used a fast presentation time of 200 ms in our current study. Our results are more consistent with the results reported by Parker et al. (2004) which indicated an absence of disgust-specific impairments in OCD patients when shorter presentation times were used (1000 ms onset), except in the case of one specific OCD patient. As these authors state in their study, OCD include a wide range of obsessive/compulsive disorders and it is possible that in some, disgust might be overestimated (for instance, in obsession with cleanliness) whereas others might cause an underestimation of this emotional feeling (for instance, collection or symmetry obsessions). Our current data point to a wide range of OCD disorders (Table 6). However, similar data have not been reported in previous studies and new experiments must be performed to test the possibility that a specific disorder affecting the emotion of disgust could be related to specific OCD. Another complementary hypothesis that should be examined in future research is that TS patients might compensate for their emotional deficit by means of a more precise perceptual analysis when longer presentation ranges are used. Although this is a *post hoc* hypothesis, this type of compensation process may explain the reversal of the EFE categorization rate for short (mainly based on spontaneous emotional feeling) vs. long (mainly based on perceptual analysis) presentation ranges.

**Table 6:** **Frequency tables for TS’s current compulsions and obsessions**.

	Without OCB	With OCB
**CURRENT COMPULSIONS**		
Cleaning/washing	0.00	37.04
Checking	1.85	14.81
Repetition	0.00	27.78
Count	11.11	22.22
Ordering/order	3.70	37.04
Collection	0.00	44.44
Others	3.70	17.78
**CURRENT OBSESSIONS**		
Aggressivity	1.59	14.29
Contamination	0.00	11.11
Sexual	0.00	11.11
Collection, accumulation	11.11	0.00
Religious	0.00	0.00
Symmetry, accuracy, order	1.85	20.37
Others	4.76	23.02
Physical	0.00	18.52

*OCD, obsessive-compulsive behaviors*.

Finally, we did not observe any difference between TS patients and MHC participants on LSF faces. It therefore seems that emotional impairments in TS may not be due to a deficit at the level of subcortical structures. One theoretical interpretation of our findings is that a potential hyperdopaminergy of subcortical structures (Albin and Mink, 2006) might compensate for a peripheral deficit induced by tic-related motor disturbances in TS. A dopaminergic hypersensitivity of the type proposed by Delfs and Kelley (1990) and Singer and Walkup (1991) or Wong et al. (1997) may have important consequences for emotional processes that take place at the level of the basal ganglia. For instance, the pallidum, a subcortical neural structure potentially involved in TS (Albin and Mink, 2006), plays a role not only in motor, but also in associative and limbic processes (Yelnik et al., 2007). It is tempting to assume that hypersensitivity in the subcortical structures thought to be involved in TS could exacerbate emotional processes at this level and consequently result in an absence of EFE deficits for LSF information. Of course, this question is as yet unresolved and the assumption stated here will have to be confirmed by further behavioral and neuroimaging experiments which differentiate between the activities of central and peripheral processes. On the basis of our current behavioral data, we can assume that similar patterns of neural activation will be found in TS and MHC for LSF faces but that there will be significant differences at the level of temporal cortical areas between TS and MHC for HSF faces.

## Conclusion

The current study indicates that the rapid visual presentation paradigm makes it possible to identify various EFE deficits (at the level of the emotions of anger and surprise) that are not revealed by the standard visual presentation procedure previously reported in the literature (Sprengelmeyer et al., 1997; Parker et al., 2004). As shown by Adolphs et al. (2005), patients with a clear deficit in their emotional *feeling* for an emotion are nonetheless able to use perceptual strategies to recognize the corresponding EFE by focusing on the cues that enable them to perform the task. We assume that emotional deficits in TS may have been hidden in previous studies due to the use of this type of perceptual analysis of the stimuli. Our study represents a first step toward more implicit measures of affective disorders in TS. However, further studies will have to build on and confirm these results using experimental designs that make it possible to detect emotional disorders independently of perceptual strategies, for example on the basis of affective priming or Rapid Serial Visual Presentation (Vermeulen et al., 2009a). Moreover, the spatial frequency analysis performed in our study suggests that emotional deficit in TS probably lies at the level of temporal cortical areas (or downstream neural structures) that are exclusively dedicated to the processing of HSF visual information and are potentially involved in embodiment processes (Niedenthal et al., 2010). Further studies involving a combination of EMG, behavioral, and neuroimaging experiments will need to differentiate accurately between the roles of central and peripheral disorders in this type of emotional deficit.

## Conflict of Interest Statement

The authors declare that the research was conducted in the absence of any commercial or financial relationships that could be construed as a potential conflict of interest.
